# Byssus Structure and Protein Composition in the Highly Invasive Fouling Mussel *Limnoperna fortunei*

**DOI:** 10.3389/fphys.2018.00418

**Published:** 2018-04-16

**Authors:** Shiguo Li, Zhiqiang Xia, Yiyong Chen, Yangchun Gao, Aibin Zhan

**Affiliations:** ^1^Research Center for Eco-Environmental Sciences, Chinese Academy of Sciences, Beijing, China; ^2^Department of Biological Sciences, Great Lakes Institute for Environmental Research, University of Windsor, Windsor, ON, Canada; ^3^College of Resources and Environment, University of Chinese Academy of Sciences, Chinese Academy of Sciences, Beijing, China

**Keywords:** *Limnoperna fortunei*, biofouling, foot protein, byssus adhesion, proteome, transcriptome, ultrastructure, metal ion

## Abstract

Biofouling mediated by byssus adhesion in invasive bivalves has become a global environmental problem in aquatic ecosystems, resulting in negative ecological and economic consequences. Previous studies suggested that mechanisms responsible for byssus adhesion largely vary among bivalves, but it is poorly understood in freshwater species. Understanding of byssus structure and protein composition is the prerequisite for revealing these mechanisms. Here, we used multiple methods, including scanning electron microscope, liquid chromatography–tandem mass spectrometry, transcriptome sequencing, real-time quantitative PCR, inductively coupled plasma mass spectrometry, to investigate structure, and protein composition of byssus in the highly invasive freshwater mussel *Limnoperna fortunei*. The results indicated that the structure characteristics of adhesive plaque, proximal and distal threads were conducive to byssus adhesion, contributing to the high biofouling capacity of this species. The 3,4-dihydroxyphenyl-α-alanine (Dopa) is a major post-transnationally modification in *L. fortunei* byssus. We identified 16 representative foot proteins with typical repetitive motifs and conserved domains by integrating transcriptomic and proteomic approaches. In these proteins, Lfbp-1, Lffp-2, and Lfbp-3 were specially located in foot tissue and highly expressed in the rapid byssus formation period, suggesting the involvement of these foot proteins in byssus production and adhesion. Multiple metal irons, including Ca^2+^, Mg^2+^, Zn^2+^, Al^3+^, and Fe^3+^, were abundant in both foot tissue and byssal thread. The heavy metals in these irons may be directly accumulated by *L. fortunei* from surrounding environments. Nevertheless, some metal ions (e.g., Ca^2+^) corresponded well with amino acid preferences of *L. fortunei* foot proteins, suggesting functional roles of these metal ions by interacting with foot proteins in byssus adhesion. Overall, this study provides structural and molecular bases of adhesive mechanisms of byssus in *L. fortunei*, and findings here are expected to develop strategies against biofouling by freshwater organisms.

## Introduction

Adhesion is the major process to form a sessile lifestyle in numerous aquatic species. Bivalve, one of the representative taxonomic groups that have strong adhesive abilities (Babarro and Comeau, 2014; Flammang et al., 2016; Kamino, 2016), can adhere to a variety of hard substrates under wet conditions with byssal threads secreted by foot glands (Waite, 2017). Byssus offers promising performance and potential to inspire underwater adhesive (Ahn, 2017), while byssus adhesion is a fundamental cause of aquatic biofouling and invasions. Large-scale biofouling, especially formed by highly invasive bivalves, poses serious threats to aquatic ecosystems, aquaculture facilities, and maritime industries (Amini et al., 2017). Such biofouling can cause corrosion of underwater facilities, water pollution, and changes in aquatic ecosystems, thus resulting in significantly ecological pollution and economic impacts (Amini et al., 2017). Therefore, deep understanding of byssus adhesion in bivalves is crucial for effectively mitigating these negative environmental impacts induced by fouling organisms, particularly by aquatic invasive species.

Byssus adhesion has been well-studied in marine mussels, especially in the genus *Mytilus*, revealing the structural characteristics of byssus and the roles of foot proteins and metal ions in byssus adhesion (Waite and Tanzer, 1981; Benedict and Waite, 1986; Waite, 1986; Zhao and Waite, 2006; Hwang et al., 2010; Yu et al., 2011; Wei et al., 2013). Marine mussel byssus is composed of proteinaceous threads, including distal thread, proximal thread, and adhesive plaque. The byssal thread is surrounded by a protective cuticle layer and supported by a collagen fiber core (Holten-Andersen et al., 2007, 2009b; Reddy and Yang, 2015). This well-structured byssus is mainly maintained by foot proteins secreted by foot glands (Wei et al., 2013). More than ten foot proteins, including collagens (preCol-NG, preCol-C, and preCol-D), thread matrix proteins (Ptmp and Tmp), and mussel foot proteins (Mfp-1-Mfp-6), have been isolated and identified from marine mussels (Waite, 2017). Mfp-1, which is mainly distributed on the surface of byssal thread, plays a protective function. Mfp-3, Mfp-5, and Mfp-6 are located in the underside of adhesive plaque, participating in byssal adhesion. Both Mfp-2 and Mfp-4 connect adhesive plaque and byssal thread and are involved in thread connection. Post-transnationally modified amino acid residues such as L-β-3,4-dihydroxyphenyl-α-alanine (Dopa) are enriched in foot proteins, contributing to byssal cohesive and adhesive interactions by formatting cross-links (Nicklisch and Waite, 2012). Furthermore, metal ions such as Ca^2+^ and Fe^3+^ bind to and interact with specific amino acids in adhesion-related proteins, playing critical roles in protein self-assembly, structure maintenance, and Dopa cross-linking (Holten-Andersen et al., 2009a; Seguin-Heine et al., 2014; Liu et al., 2015; Li S. G. et al., 2017). These results comprehensively show that byssus adhesion is a multi-level complex process, and elements at different levels (e.g., structure, protein, and metal ion) play their crucial roles in such a complex process.

Interestingly, numerous studies showed that elements involved at different levels largely varied among species, suggesting that species- and/or taxonomic group-specific mechanisms of byssus adhesion should be deeply investigated in organisms of interest. For example, a study on the scallop *Chlamys farreri* identified seven byssal proteins (Sbp1-Sbp7) and their associated genes, and intriguingly such proteins showed limited homologies to the currently known ones in mussels (Miao et al., 2015). Genomic analyses for this species highlighted that the expanded tyrosinases enabled rapid byssal formation (Guerette et al., 2013; Li Y. L. et al., 2017). Among fourteen byssal proteins identified from the pearl oyster *Pinctada fucata*, none of them could match Mfps or Sbps (Liu et al., 2015). Regarding the structure of byssus, a great number of nano-cavities were found in the inner core of distal thread in *P. fucata*, while such a structure was not observed in marine mussels and scallops (Liu et al., 2015; Reddy and Yang, 2015). Moreover, differences in both the type of metal ions and contents were also found among these marine bivalves (Seguin-Heine et al., 2014; Liu et al., 2015; Li S. G. et al., 2017).

Compared with marine species, byssus has been rarely studied in freshwater bivalves, though its adhesive mechanisms have been suggested to be species-specific. For example, two former studies identified a total of 12 novel foot proteins (Dpfp1-Dpfp12) from the freshwater mussel *Dreissena polymorpha*, showing great difference from foot proteins in marine bivalves. Ultrastructure difference was also found between *D. polymorpha* and marine mussels (Xu and Faisal, 2008; Gilbert and Sone, 2010; Farsad and Sone, 2012; Gantayet et al., 2013). So far, reasons are still not clear for such a high level of differences in adhesive mechanisms among bivalves, particularly between marine and freshwater species, largely owing to the lack of available information in freshwater species. It is therefore of great necessity to study byssus structure and protein composition in more freshwater counterparts, particularly under the circumstances that many freshwater bivalves have largely expanded their distribution ranges and caused severe biofouling problems in both native and invasive ranges, such as the golden mussel *Limnoperna fortunei* and *Dreissena* mussels (Zhan et al., 2012).

The golden mussel *L. fortunei* is a small-sized freshwater bivalve in the family Mytilidae with strong invasiveness. It is native to Southeast Asia and has invaded many freshwater ecosystems in Asia high-latitude regions and South America since 1990s (Zhan et al., 2012; Xia et al., 2017). *L. fortunei* often forms dense mussel beds by cross connecting among individuals or adhering to submersed substrates, causing severe problems such as pipe occlusion, water flow reduction, filter, and heat exchanger blocking and concrete corrosion in natural structures and manmade facilities (Boltovskoy et al., 2015). Such a severe biofouling problem has caused subsequent economic damages (Ohkawa et al., 1999a; Boltovskoy et al., 2015). In addition, due to its powerful filtering capacity, the invasion, and biofouling of *L. fortunei* can alter the structure and stability of freshwater ecosystems (Welladsen et al., 2011; Boltovskoy et al., 2015; Xu et al., 2015). A few studies have tried to investigate foot structure and proteins of *L. fortunei*, and to explore potential mechanisms of byssus adhesion in such a high-impact species. For example, microscopic observation revealed that secretory cells in the groove on ventral portion of foot tissue in *L. fortunei* could secret mucous to produce byssal threads, indicating a cytological mechanism of byssus formation (Andrade et al., 2015). Two candidate glue proteins (i.e., Lffp-1 and Lffp-2) were successfully isolated from *L. fortunei* foot tissue (Ohkawa et al., 1999b; Ohkawa and Nomura, 2015), while only one foot protein gene, *LfFP-2*, has been fully sequenced (Uliano-Silva et al., 2014). Despite such research progress, the mechanism of byssus adhesion at multiple levels such as byssus structure, protein composition and metal ion in *L. fortunei* remains largely unexploited. Such a gap hinders not only better understanding of the difference in byssus adhesion between marine and freshwater bivalves, but also exploring prevention strategies against biofouling caused by this species.

Recently, the dual transcriptomic and proteomic approach has emerged as the best way to identify novel adhesion-related proteins in animals (Guerette et al., 2013; Hennebert et al., 2015). In this study, multiple analyses were conducted to study byssus in the golden mussel *L. fortunei*, from structural characteristic, protein composition and gene expression to metal ions that may participate in crucial processes of byssus formation and adhesion. Specifically, inductively coupled plasma mass spectrometry (ICP-MS) was employed to determine metal ion content and liquid chromatography–tandem mass spectrometry (LC-MS/MS), transcriptome sequencing, and real-time quantitative PCR (RT-qPCR) to identify foot proteins in foot tissue and byssal thread. The interactions of foot proteins and metal ions were demonstrated by analyzing amino acid preference and metal ion contents. Byssus ultrastructure, which is primarily maintained by interactions between foot proteins and metal ions, was observed using light microscope (LM) and scanning electron microscope (SEM). Nitroblue tetrazolium (NBT)/Glycinate staining were used to analyze the existence of Dopa modification in byssus. This structure and protein composition study is expected to provide significant backgrounds for deeply understanding the underlying mechanisms of byssus adhesion in the highly invasive *L. fortunei*.

## Materials and methods

### Sample collection and experimental design

Adult golden mussels *L. fortunei* with medium size (1.5–2.0 cm) were collected from Shisanling Reservoir, Beijing, China (40°15′ N, 116°15′ E) in June 2017. Mussels were transported to laboratory within 2 h after collection under low temperature (~15°C). Before downstream analyses, all mussels were cultured in a circulating aquarium at 20 ± 1°C for 1 week and daily fed with pure culture of algae *Chlorella* sp.

Multiple methods on foot tissue and byssal thread were used to clarify the mechanism of byssus adhesion in *L. fortunei*. To prepare newly secreted byssal thread, live mussels were carefully cut from original byssal threads and placed on glass slides to allow them to re-attach. The most important compositions involved in byssus adhesion, foot protein and metal ion were identified and further analyzed in foot and newly secreted byssus. Byssus ultrastructure associated with these two compositions was observed and characterized.

Foot tissue was dissected from live mussels using disinfected surgical blades, washed three times with double deionized water (ddH_2_O), rapidly frozen with liquid nitrogen and preserved at −80°C. Meanwhile, whole byssal threads, including proximal thread, distal thread, and adhesive plaque, were collected from the same mussels, washed with ddH_2_O and preserved at −80°C. The foot tissues and byssal threads from 30 mussels were pooled together as a mixed sample for total RNA and protein extraction.

Newly secreted byssal threads with whole structure were randomly dissected from 10 individuals. Byssal thread samples were then washed three times with ddH_2_O, placed in a petri dish and preserved at 4°C for morphological observation. In addition, byssal threads with whole structure were dissected, washed three times with ddH_2_O water, air-dried at room temperature and preserved in a sealed desiccator. Byssal threads from 30 individuals were pooled together as a biological replicate and three replicates were used for metal ion determination.

Foot, mantle, gonads, visceral mass, adductor muscle, and gill were collected, washed three times with RNase-free water, immediately frozen with liquid nitrogen and preserved at −80°C for tissue-specific gene expression analysis. In addition, byssal threads from 150 mussels were carefully removed and these mussels were randomly divided into three 50-individual groups. Each group was then cultured in a 20 L aerated tank under 20 ± 1°C for 7 days. The number of newly produced byssus was recorded daily. Foot tissues were collected from 10 individuals per tank on the 1st, 3rd, 5, and 7th day, respectively. These foot samples were washed three times with RNase-free water, immediately frozen with liquid nitrogen and preserved at −80°C for expression analysis for foot protein genes. Foot tissues from 10 individuals were pooled together as a biological replicate and three replicates were used for the above gene expression analyses.

### Light microscopy (LM) and scanning electron microscopy (SEM)

To analyze byssus structure, byssus samples were observed with a Light Microscope (CX41, Olympus, Tokyo, Japan). The proximal thread, distal thread, and adhesive plaque of each sample were photographed and recorded with CellSens Standard (Olympus, Tokyo, Japan). Meanwhile, byssal threads were sputter-coated with gold particles for 60 s in Rotary Pumped Sputter Coater (E1010, Hitachi, Tokyo, Japan) and observed using a Scanning Electron Microscope (SEM, FEI Quanta 200, Netherlands) following the previously described method (Li S. G. et al., 2017).

### Dopa staining analysis

To confirm the existence of *post-translational* modification Dopa in byssus of *L. fortunei*, byssal thread and adhesive plaque samples were stained with nitroblue tetrazolium (NBT)/Glycinate following Qin et al. (2016). Briefly, the golden mussel samples were raised in an aquarium with glass slides at the bottom. After adhesion on the slides, the byssus was extracted from mussel body and then scraped away from slides. The integrated byssus samples were secreted into slices with five micron thick using the classical paraffin section method. The obtained byssus slices were subsequently stained with NBT/Glycinate solution (0.24 mmol/L NBT in 2 mol/L potassium glycinate, pH 10) in the dark for 2 h at 25°C. After staining, the slides were washed with sterilized water for three times and then observed using a light microscope (CX41, Olympus, Tokyo, Japan). Three slides were considered as three biological replicates and at least 10 byssal threads were observed for each replicate.

### *De novo* transcriptome sequencing

To identify adhesion-related genes, total RNA of foot tissue samples was extracted using mirVana miRNA Isolation Kit (Ambion Inc, Austin, Tex, USA) following the manufacturer's instructions. RNA integrity was evaluated using Agilent 2100 Bioanalyzer (Agilent Technologies, Santa Clara, CA, USA). Total RNA with integrity number (RIN) ≥ 7 was used for downstream analyses. A cDNA library was constructed using TruSeq Stranded mRNA LTSample Prep Kit (Illumina, San Diego, CA, USA) according to manufacturer's instructions and sequenced on the Illumina Sequencing Platform HiSeqTM 2500 (Illumina, San Diego, CA, USA). The sequenced results were spliced by TRINITY software (Grabherr et al., 2011) with paired-end method. TGICL software was used to remove redundancy and get a set of final unigene as reference sequences. Raw data of *L. fortunei* foot transcriptome has been deposited into Sequence Read Archive (SRA) in NCBI (accession number SRP125019). These obtained sequences were subsequently submitted to Basic Local Alignment Search Tool (BLAST) against NR database (ftp://ftp.ncbi.nih.gov/blast/db) and Swiss-Prot database (http://www.uniprot.org/downloads) for functional annotation. A cutoff E-value ≤ 1.0e^−5^ was used to identify the most representative annotation. Clusters of Orthologous Groups for Eukaryotic Complete Genomes (COG) and Kyoto Encyclopedia of Genes and Genomes (KEGG) annotation were also performed using previously described methods (Hou et al., 2017; Li S. G. et al., 2017) with the cutoff E-value ≤ 1.0e^−5^. Typical unigenes with high homologies to the reported adhesion-related proteins in this transcriptome were selected and compared with other bivalve species using homologous alignment methods (Guerette et al., 2013; DeMartini et al., 2017). Homologous alignments for foot proteins were conducted using BioEdit version 7.2.5 (Hall, 1999) and Jalview online bioinformatics tool (http://www.jalview.org/). Repetitive motifs were predicted by RADAR tool (https://www.ebi.ac.uk/Tools/pfa/radar/) and conserved domains were predicted by PROSITE tool (http://prosite.expasy.org/).

### Protein extraction and mass spectrometry analysis

To analyze protein composition of byssal thread, foot tissue, and byssal thread samples were grinded in liquid nitrogen. Total foot and byssal proteins were extracted using the acetone precipitation method. Briefly, the grinded sample was put into a centrifuge tube, added cool acetone containing 10% trichloroacetic acid (TCA) and then precipitated at −20°C for 2 h. The precipitation was collected by centrifugation at 20,000 g for 10 min at 15°C, washed with 100% acetone and precipitated for another 30 min at −20°C. This precipitation protocol was repeated for three times. Subsequently, 500 μL L3 solution (1 mM PMSF, 2 mM EDTA and 10 mM DTT) was added into the tube following 5 min ultrasound lysis. The ultrasonic program included pulse on for 2 s and pulse off for 3 s at 180 W. The resulted sample was then 20,000 g centrifuged at 15°C for 30 min and supernatant was collected. The Bradford method was used for the protein quantitation. Specifically, 20 μg extracted proteins were separated by routine Sodium Dodecyl Sulfate Polyacrylamide Gel Electrophoresis (SDS-PAGE, 12%) and stained with Coomassie Blue R-250.

Candidate regions were selected from the SDS-PAGE gel and digested by trypsin using the acetonitrile and ammonium bicarbonate method. The digested peptides were further identified by Liquid Chromatography–tandem Mass Spectrometry (LC-MS/MS) using Ultimate 3000 RSLCnano System coupled to Q Exactive Mass Spectrometer (Thermo Scientific, Waltham, USA). The obtained data was further searched against Mollusca UniProt database (http://www.uniprot.org/taxonomy/6447) and the unigene library was generated from the foot transcriptome using MASCOT 2.3.02 (Matrix Science, London, UK). Experimental operation and data analysis were based on the methods described in our previous studies (Li et al., 2016; Li S. G. et al., 2017). The results with Unique Peptide ≥ 2, Coverage ≥ 30 and Score ≥ 100 were considered as representative foot proteins in foot and byssus. Subsequently, the results synchronously presented in foot transcriptome, foot proteome and byssus proteome were screened and considered as the most representative foot proteins. Amino acid compositions of these proteins were analyzed using ProtParam tool (http://web.expasy.org/protparam/). Repetitive motifs were predicted by RADAR tool (https://www.ebi.ac.uk/Tools/pfa/radar/) and domains were predicted by PROSITE tool (http://prosite.expasy.org/).

### Real-time quantitative PCR (RT-qPCR)

Expressions of foot protein genes were analyzed using the RT-qPCR method. Foot tissue samples were grinded in liquid nitrogen. Total RNA isolation, purification and quantification were conducted using methods by Li S. G. et al. (2017). PrimeScript™ RT Reagent Kit (Takara, Tokyo, Japan) was used to synthesize cDNA following the manufacturer's instructions. A 20-μL reaction containing 0.4 μM of each primer, 1.0 μL of cDNA, and AceQ qPCR SYBR® Green Master Mix (Vazyme, Nanjing, China) was carried out on a LightCycler® 96 System (Roche Diagnostics, Mannheim, Germany). Thermal program included 1 cycle of 95°C for 600 s; 40 cycles of 95°C for 10 s and 60°C for 30 s; 1 cycle of 95°C for 10 s, 65°C for 30 s and 95°C for 10 s. The *LfACTIN* gene was used as the internal reference (Uliano-Silva et al., 2014). For tissue-specific analyses, the adductor muscle was used as the control tissue. For gene expression profile analysis, foot tissues from pre-cultured mussels were used as the day 0 control. Relative expression levels of these genes were calculated by the 2^−ΔΔCT^ method (Livak and Schmittgen, 2001). Specific primers for all the genes (Table S1) were designed using Primer3 version 4.1.0 (http://primer3.ut.ee/).

### Inductively coupled plasma mass spectrometry (ICP-MS)

To analyze metal ion composition and content, byssal thread, and foot tissue samples were firstly hydrolyzed in a mix of nitric acid (HNO_3_) and hydrogen peroxide solution (H_2_O_2_, 30%) at 90°C for 3 h, respectively. Metal ion contents were determined using Inductively Coupled Plasma Mass Spectrometry (ICP-MS, Agilent 7500c, New Castle, DE, USA) with a micro-nebulizer following the protocol by Li S. G. et al. (2017) The determined metal elements included calcium, magnesium aluminum, iron, zinc, manganese, lead, copper, mercury, boron, molybdenum, nickel, and molybdenum. These metal ions were suggested to play key roles in byssus performance in bivalves (Seguin-Heine et al., 2014; Liu et al., 2015). Metal ion contents were presented as mg metal·g tissue^−1^ for foot tissues and mg metal·g byssus^−1^ for byssal threads. The percentage of each metal ion in the total contents of metal ions in both foot tissue and byssal thread samples were also calculated.

### Statistical analysis

Statistical analyses of data were obtained from three biological replicates and expressed as mean ± standard deviation (SD). Differences in gene expression levels and byssus numbers were accessed using one-way ANOVA with the significance level at ^*^p < 0.05. Micrographs were processed in Adobe Photoshop CS4 11.0 software and figures were drawn using SigmaPlot version 12.5 (Systat Software, San Jose, CA, USA).

## Results

### Byssus morphology and structure

**Figure 1:**
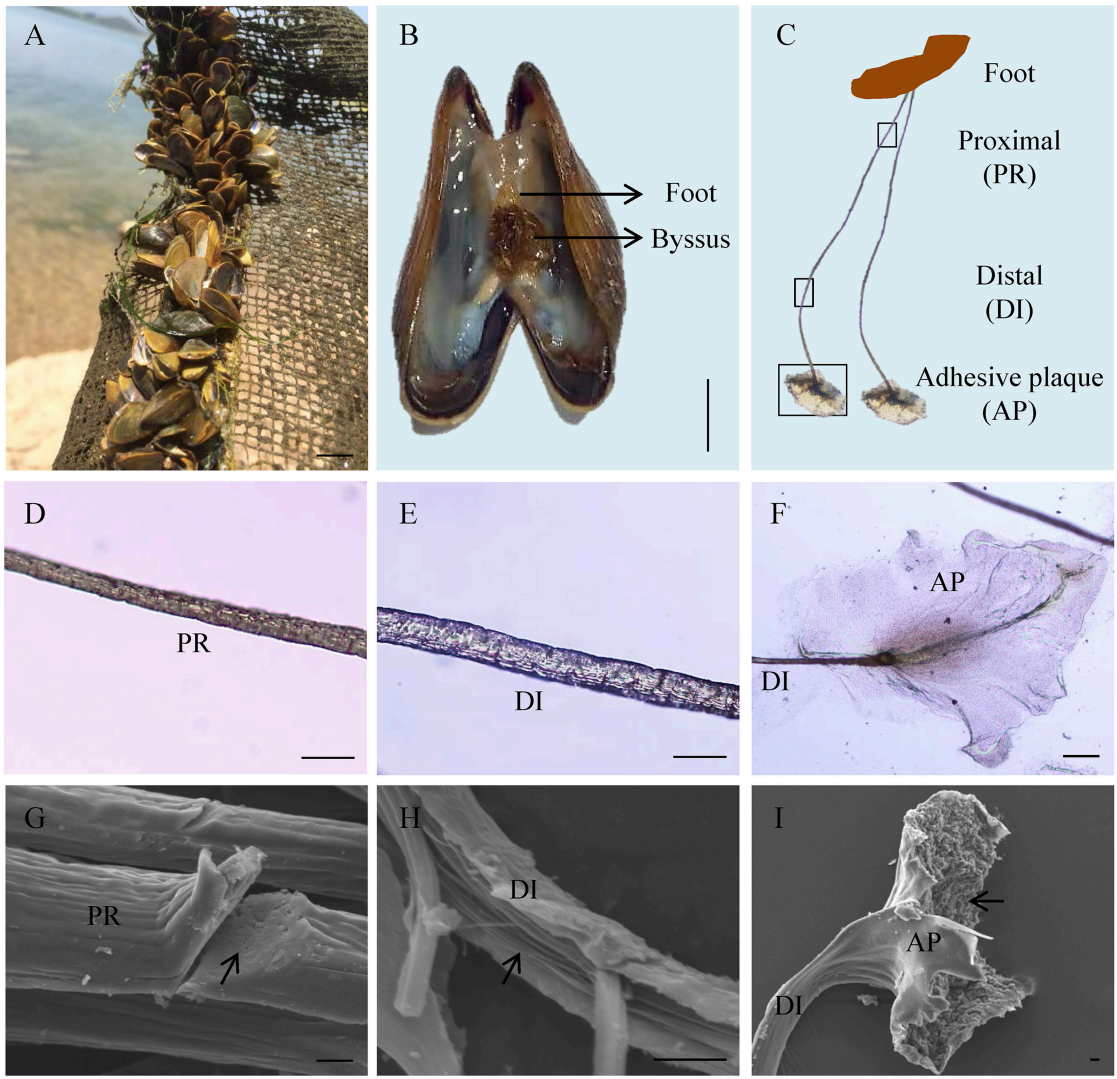
Byssus morphology and structure of the golden mussel *Limnoperna fortunei*. **(A)** *L. fortunei* living in natural freshwater. **(B)** Photograph showing the position and morphology of foot and byssus. **(C)** The structure of byssal threads. PR, Proximal thread; DI, Distal thread; AP, Adhesive plaque. The black boxes indicate the observation regions for **(D–F)**. **(D)** Light photograph for the PR region. **(E)** Light photograph for the DI region. **(F)** Light photograph for the AP region. **(G)** Scanning electron micrograph for the PR region. The arrow indicates the compact ultrastructure of PR. **(H)** Scanning electron micrograph for the DI region. The arrow indicates the fibroid ultrastructure of DI. **(I)** Scanning electron micrograph for the AP region. The arrow indicates the rough underside of AP facing to substratum. Bar scale is 1 cm in **(A,B)**, 50 μm in **(D–F)**, and 10 μm in **(G–I)**.

Adult *L. fortunei* could robustly adhere on underwater substrates *via* byssus in natural environments (Figure 1A). The byssus, which was secreted by foot tissue (Figure 1B), was 1.0–1.5 cm in length and could be divided into three parts: proximal thread, distal thread, and adhesive plaque (Figure 1C). Diameter of a byssal thread increased from proximal (15–20 μm) to distal end (25–30 μm, Figures 1D,E). At the distal end, an elliptical adhesive plaque with dramatically larger surface area (around 0.08 cm^2^) allowed *L. fortunei* to fully attach to hard substrates (Figure 1F). SEM observation illustrated two distinct structures of a byssal thread: an outer cuticle and an inner fibrous core. The cuticle was smooth and fibers were closely aligned in the proximal thread, forming a compact structure (Figure 1G). Unlike proximal thread, the distal thread was a loosely organized structure with distinct boundary among inner filamentary fibers (Figure 1H). The cuticle of the adhesive plaque was smooth, while the underside attaching to substratum was rough with numerous cavities distributed along the thread (Figure 1I).

### Dopa staining analysis

**Figure 2:**
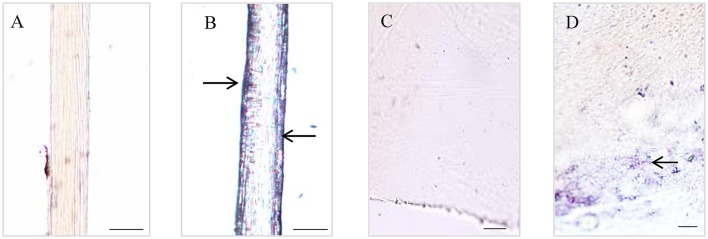
Histological staining observations on different parts of *Limnoperna fortunei* byssus. Light micrographs of byssal thread longitudinal section before **(A)** and after **(B)** nitroblue tetrazolium (NBT)/Glycinate staining. Light micrographs of adhesive plaque transverse section before **(C)** and after **(D)** NBT/Glycinate staining. The arrows indicate the distribution regions of Dopa-containing proteins stained by NBT/Glycinate. Bar scale is 20 μm in **(A**,**B)**, and 10 μm in **(C,D)**.

The Dopa-containing proteins stained by NBT/Glycinate had liner distributions in the longitudinal section of byssal thread (Figure 2) and were located at the outer sheath (Figures 2A,B). However, in adhesive plaque they were flocculent structures and located at the bottom of adhesive plaque transverse section (Figures 2C,D). These results suggest the potential locations of Dopa-containing foot proteins in different parts of byssus.

### Transcriptome sequencing and adhesion-related gene screening

A total of 73,231,128 nt raw data and 72,686,438 clean sequence reads were obtained from foot tissues, and the percentage of base number with Phred > 30 (Q30) in total bases is 92.89% (Table S2), yielding 92,951 unigenes with an average length of 681 bp and a N50 of 946 bp (Table S3 and Figure S1). A total of 26,262 (28.25%) and 21,624 (23.26%) unigenes were annotated to NR and Swiss-Prot database, respectively. Due to the lack of availability of freshwater species, the retrieved species were mainly confined in marine invertebrates such as *Crassostrea gigas, Lottia gigantea, Lingula anatina, Aaplysia californica*, and *Octopus bimaculoides* in NR database (E-value ≤ 1.0e-5, Figure S2) and vertebrates such as *Homo sapiens, Mus musculus, Rattus norvegicus*, and *Danio rerio* in Swiss-Prot database (E-value ≤ 1.0e-5, Figure S3).

A total of 17,871 (19.23%) unigenes were assigned to COG terms (Figure S4 and Table S4). The most representative terms for COG analysis were “Post-transcriptional modification, protein turnover, chaperone,” “General function prediction only,” “Function unknown,” and “Signal transduction mechanism.” Meanwhile, a total of 9,368 unigenes (10.08%) were assigned to KEGG terms (Figure S5 and Table S5). The most representative terms for KEGG analysis were “Transport and catabolism” and “Cell growth and death” in “Cellular process,” “Signal transduction” in “Environmental information processing” and “Nervous system,” “Immune system” and “Endocrine system” in “Organismal system”.

**Figure 3:**
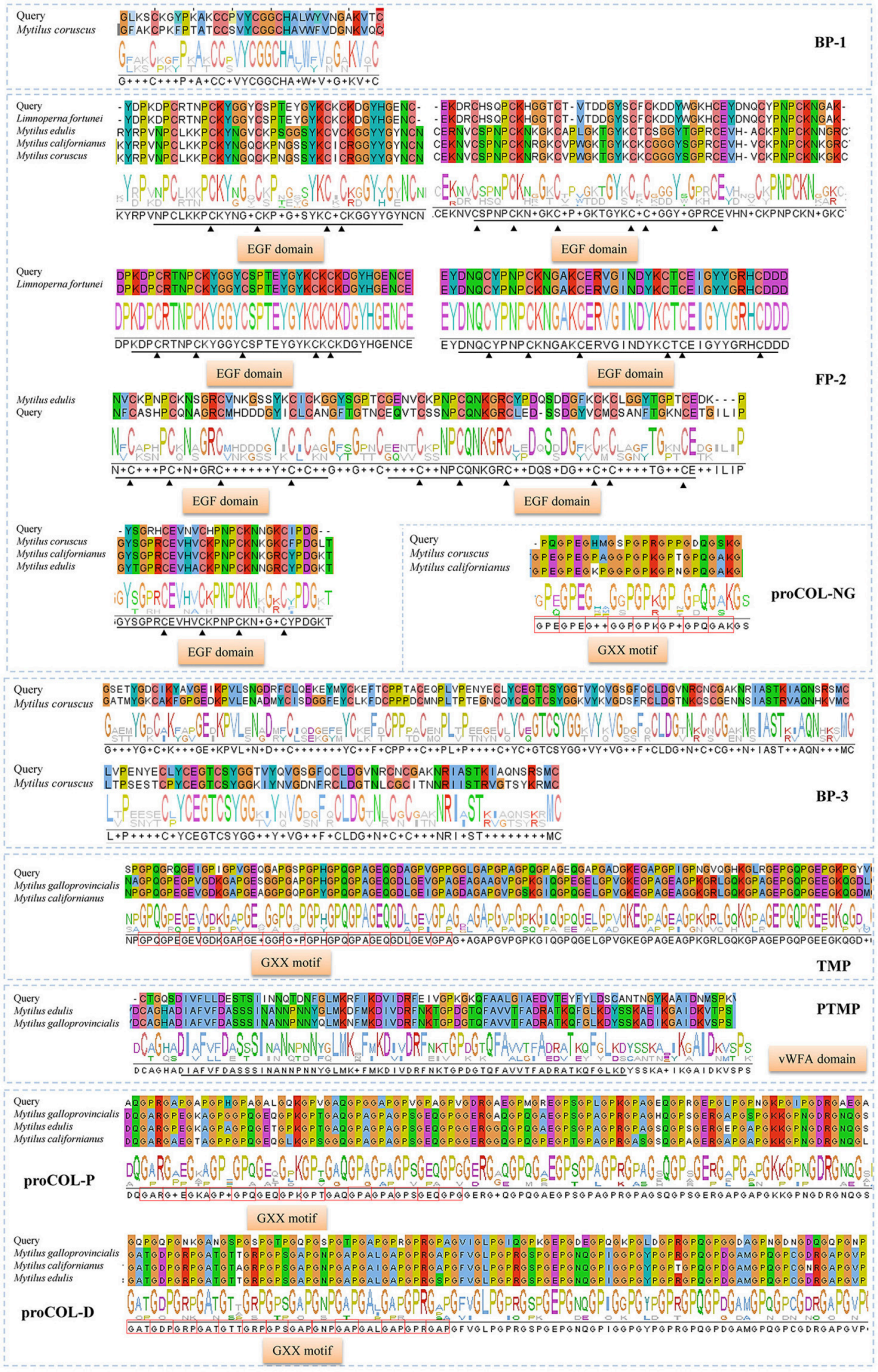
Homologous alignments of foot protein genes identified from foot transcriptome of *Limnoperna fortunei*. Alignment result for each gene contains similarity regions of amino acids (shadow) and consensus logo (below the shadow). The regions marked by black straight lines and triangles are conserved EGF domains and the red boxes indicate GXX motifs. The amino acid sequences used for these alignments are as follows. Bp-1: *Mytilus coruscus* [AKI87984.1]; Fp-2: *Limnoperna fortunei* [BAO74176.1], *Mytilus edulis* [AAX23970.1], *Mytilus californianus* [AST36139.1] and *Mytilus coruscus* [ALA16015.1]; Bp-3: *Mytilus coruscus* [AKI87986.1]; proCOL-NG: *Mytilus coruscus* [ALA16018.1] and *Mytilus californianus* [ABW90433.1]; proCOL-D: *Mytilus galloprovincialis* [AAM34601.1], *Mytilus californianus* [ABW90432.1] and *Mytilus edulis* [AAB96638.1]; proCOL-P: *Mytilus galloprovincialis* [AAM34600.1], *Mytilus edulis* [AAB80719.1], and *Mytilus californianus* [ABW90434.1]; TMP: *Mytilus galloprovincialis* [AHI47022.1] and *Mytilus californianus* [ABW90433.1]; PTMP: *Mytilus edulis* [AAL83537.1] and *Mytilus galloprovincialis* [AAL17974.1].

In total, eight typical adhesion-related genes, which contained 12 unigenes, were obtained from the foot transcriptome (Table 1). These genes encoded foot/byssal proteins (*FP-1, FP-2, FP-3*), thread matrix protein (*TMP*), proximal thread matrix protein (*PTMP*), and precollagen (*preCOL-NG, preCOL-P*, and *preCOL-D*). These adhesion-related unigenes were well-annotated to corresponding homologs in marine mussels *M. coruscus, M. galloprovincialis*, and *M. edulis* with an average identity of 45.25%. Alignment analysis indicated that the foot proteins encoded by these genes in *L. fortunei* contained typical domains and motifs of known mussel foot proteins, such as EGF domain in foot protein 2, vWFA domain in proximal thread matrix protein and GXX motif in thread matrix protein and precollagens (Figure 3).

**Table 1:** Typical adhesion-related genes in foot transcriptome of the golden mussel *Limnoperna fortunei*.

**Unigene**	**Symbol**	**Length (bp)**	**Description**	**E-value**	**Identity (Amino acid)**	**Domain / motif**	**Swiss-id**
comp98491_c0_seq1	BP-1	510	|AKI87984.1|byssal protein-1 [*Mytilus coruscus*]	7.0e-35	(70/135) 51.85%	—	sp|P33450|FAT_DROME
comp113423_c0_seq1	FP-2	363	|BAO74176.1|putative foot protein-2 [*Limnoperna fortunei*]	1.0e-19	(106/108) 98.15%	EGF	sp|Q14517|FAT1_HUMAN
comp117406_c1_seq2	FP-2	524	|BAO74176.1|putative foot protein-2 [*Limnoperna fortunei*]	5.0e-26	(132/133) 99.25%	EGF	sp|Q2PZL6|FAT4_MOUSE
comp155923_c0_seq1	FP-2	549	|AAX23970.1|foot protein 2 [*Mytilus edulis*]	4.0e-14	(56/140) 40.00%	EGF	sp|Q25464|FP2_MYTGA
comp119431_c0_seq1	FP-2	1111	|AAX23970.1|foot protein 2 [*Mytilus edulis*]	5.0e-88	(173/337) 51.34%	EGF	sp|Q25464|FP2_MYTGA
comp112372_c0_seq2	BP-3	1199	|AKI87986.1|byssal protein-3 [*Mytilus coruscus*]	3.0e-34	(55/129) 42.64%	—	sp|P49013|FBP3_STRPU
comp102200_c0_seq2	BP-3	696	|AKI87986.1|byssal protein-3 [*Mytilus coruscus*]	1.0e-24	(55/129) 42.64%	—	—
comp119176_c1_seq3	TMP	1399	|AAC33847.1|thread matrix protein [*Mytilus edulis*]	9.0e-28	(21/35) 60.00%	GXX motif	—
comp107331_c0_seq3	PTMP-1a	999	|AAL83537.1|proximal thread matrix protein 1 variant a [*Mytilus edulis*]	5.0e-12	(51/151) 33.77%	vWFA	sp|A2AX52|CO6A4_MOUSE
comp119875_c0_seq1	proCOL-NG	1571	|ALA16018.1|precollagen NG [*Mytilus coruscus*]	3.0e-12	(125/220) 56.82%	GXX motif	sp|A6H584|CO6A5_MOUSE
comp120266_c1_seq1	proCOL-P	2866	|AAM34600.1|precollagen-P [*Mytilus galloprovincialis*]	3.0e-13	(44/133) 33.08%	GXX motif	sp|Q14050|CO9A3_HUMAN
comp124514_c0_seq1	proCOL-D	2246	|AAB96638.1|precollagen D [*Mytilus edulis*]	1.0e-147	(44/131) 33.59%	GXX motif	sp|P27393|CO4A2_ASCSU

*The “—” indicates no characterized domain*.

### LC-MS/MS and foot proteins identification

Proteins were successfully extracted from foot tissue and byssal thread of *L. fortunei*. Totally, 11 clear regions with a molecular weight range of 10–150 KD were dissected from SDS-PAGE gels for foot and byssal proteins (Figure S6). LC-MS/MS analysis detected 18 byssal proteins from byssal thread (Table S6) and 81 proteins from foot tissue (Table S7), respectively. By integrating adhesion-related unigenes, foot proteins and byssal proteins, a total of 16 foot proteins were identified (Table 2, Figure S7). These proteins could be classified into four types: (1) foot/byssal proteins, such as foot protein-2 (Lffp-2), byssal protein-1 (Lfbp-1), and byssal protein-3 (Lfbp-3); (2) enzymes: such as byssal peroxidase-like proteins (type 1, 2, and 4) and protease inhibitor-like protein-D2; (3) cellular framework proteins, such as tubulin alpha chain, histone H2B, and ribosomal proteins (type S3 and S14); and (4) other associated proteins, such as tropomyosin, p-glycoprotein, heat shock protein 70 (HSP 70), and byssal HSP-like protein 1. These adhesives proteins exhibited high homologies to known foot/byssal proteins in bivalves. In addition, Ala, Asp, Gly, Glu, Leu, Ser, Thr, and Val were the most abundant amino acids. The abundances of these amino acids were between 6 and 18%. Multiple repetitive motifs were found in these foot proteins. Distinct functional domains were also predicted, such as epidermal growth factor (EGF) domain in foot protein-2, ALPHA-TUBLIN domain in tubulin alpha chain, TROPOMYOSIN domain in tropomyosin, and HSP-70 domain in heat shock protein 70.

**Table 2:** The most representative foot proteins identified from the golden mussel *Limnoperna fortunei*.

**Accession**	**Description**	**Score**	**Coverage**	**Unique Peptide**	**Amino acid composition**	**Domain**	**Matched Unigene**
O96064	Paramyosin [*Mytilus galloprovincialis*]	1746.57	30.09	19	Ala (9.43%) Gln (7.51%) Gly (13.29%) Val (11.36%) Pro (6.64%)	—	comp124156_c0_seq1
A0K0YAX7	Filament-like protein-2 [*Mytilus coruscus*]	1284.48	40.88	14	Ala (7.92%) Asp (6.78%) Glu (7.32%) Gly (8.56%) Leu (7.37%) Pro(6.21%) Ser (7.45%) Thr (7.34%) Val (6.56%)	—	comp126030_c0_seq8
K1QQ68	Tubulin alpha chain [*Crassostrea gigas*]	1010.37	41.46	2	Ala (6.42%) Asp (6.71%) Glu (7.78%) Gly (8.74%) Leu (6.11%) Pro (7.34%) Ser (7.02%) Thr (6.14%) Val (7.33%)	ALPHA-TUBULIN	comp39206_c0_seq1
Q9GZ70	Tropomyosin [*Perna viridis*]	778.09	45.07	15	Ala (7.27%) Asp (7.04%) Glu (9.29%) Gly (7.25%) Leu (6.53%) Pro (6.46%) Ser (7.52%) Thr (6.02%) Val (7.28%)	TROPOMYOSIN	comp116888_c0464_seq2
A0K0YB30	Protease inhibitor-like protein-D2 [*Mytilus coruscus*]	571.54	241.99	2	Ala (9.22%) Asp (7.31%)Glu (7.25%) Gly (6.54%) Leu (10.32%) Pro (6.12%) Ser (6.94%) Thr (9.17%)	—	comp102054_c0_seq3
F0V4B3	Byssal protein-1 [*Mytilus galloprovincialis*]	445.72	8.96	2	Ala (9.61%) Asp (6.38%) Ile (6.01%) Leu (9.63%) Ser (3.57%) Thr (8.03%) Val (7.59%)	—	comp98491_c0_seq1
A024FCK3	Putative foot protein-2 [*Limnoperna fortunei*]	439.32	37.39	2	Ala (7.10%) Arg (6.54%) Asn (9.50%) Leu (7.72%) Met (7.74%) Phe (8.28%) Pro (9.45%) Val (10.10%)	EGF	comp119431_c0_seq1
A193DU97	Byssal HSP-like protein 1 [*Mytilus coruscus*]	340.48	38.51	2	Ala (7.44%) Asp (6.91%) Glu (6.43%) Gly (10.57%) Leu (6.91%) Pro (6.85%) Val (8.52%)	—	comp126799_c1_seq1
U5Y6N6	P-glycoprotein [*Perna viridis*]	300.07	36.31	4	Arg (6.12%) Asp (7.08%) Gln (10.21%) Gly (6.11%) Leu (8.17%) Pro (6.10%) Ser (10.22%) Val (8.24%)	—	Comp43007_c0_seq1
K1P7X2	40S ribosomal protein S14 [*Crassostrea gigas*]	288.09	88.32	2	Asp (9.04%) Gln (7.67%) Glu (14.13%) Gly (12.83%) Leu (11.45%) Ser (7.73%) Val (6.43%)	—	comp106676_c0_seq1
Q6PTI3	Byssal protein-3 [*Mytilus californianus*]	191.99	10.06	2	Asn (10.31%) Glu (7.40%) Gly (11.84%) Leu (14.70%) Thr (10.31%)	—	comp112372_c0_seq2
A193DUA2	Byssal peroxidase-like protein 2 [*Mytilus coruscus*]	190.68	201.35	2	Ala (7.70%) Asp (7.67%) Glu (8.78%) Ile (9.92%) Leu (13.22%) Ser (7.71%)	AN_PEROXIDASE	comp125734_c0_seq1
K1PUQ5	Histone H2B [*Crassostrea gigas*]	180.88	35.77	4	Ala (6.77%) Asp (6.75%) Gly (10.20%) Ile (9.14%) Leu (14.78%) Lys (6.76%) Thr (8.03%) Val (9.12%)	—	comp155404_c0_seq1
V5LVI9	Heat shock protein 70 [*Vulcanidas* sp.]	178.37	37.46	2	Ala (10.90%) Asp (7.12%) Glu (10.37%) Gly (7.72%) Leu (9.31%) Lys (6.04%) Phe (6.62%) Thr (8.67%)	HSP-70	comp113462_c0_seq1
A193DUA3	Byssal peroxidase-like protein 4 [*Mytilus coruscus*]	168.44	83.59	4	Ala (12.79%) Asn (7.65%) Asp (6.42%) Glu (12.80%) Ile (11.53%) Leu (7.67%) Phe (6.42%)	—	comp119654_c0_seq1
Q70MM6	Ribosomal protein S3 [*Crassostrea gigas*]	112.65	58.18	3	Asn (9.80%) Asp (7.78%) Gly (11.79%) Leu (17.56%) Thr (11.83%)	—	comp113325_c0_seq1

*The “—” indicates no characterized domain*.

### Expression profiles of the foot protein genes

**Figure 4:**
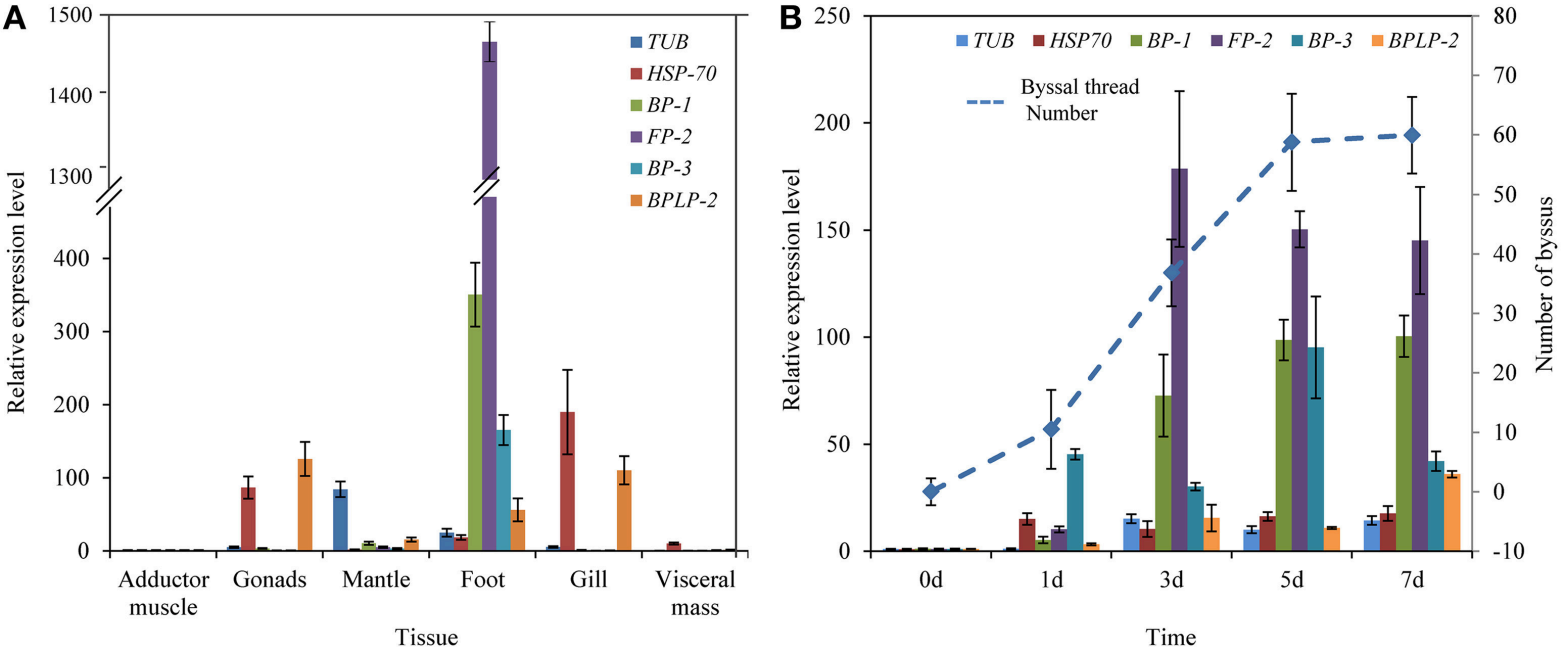
Relative expression levels of six selective foot protein genes in different tissues **(A)** and byssus formation period **(B)** in the golden mussel *Limnoperna fortunei* detected by real-time quantitative PCR. *TUB*, Tubulin alpha chain; *HSP-70*, Heat shock protein 70; *BP-1*, Byssal protein-1; *FP-2*, Putative foot protein-2; *BP-3*, Byssal protein-3; *BPLP-2*, Byssal peroxidase-like protein 2. **(B)** The line chart indicates the changes in byssal thread numbers.

RT-qPCR results indicated that the expression of selected foot protein genes was tissue-specific (Figure 4A). The foot/byssal protein genes *BP-1, FP-2*, and *BP-3* were specifically expressed in foot and their expression levels were 350.62-, 1484.25-, and 165.43-fold higher than that in adductor muscle, respectively. *TUB, HSP-70*, and *BPLP-2* also expressed in foot tissue but their expressions were more concentrated in other tissues. The relative expression levels of *TUB, HSP-70*, and *BPLP-2* were 84.38-fold higher in mantle, 190.01-fold higher in gonads and 125.89-fold higher in gonads than that in adductor muscle, respectively (*p* < 0.05). Byssal threads rapidly regenerated at early stages after removing original byssus and leveled off after 5 days (Figure 4B), indicating a platform period for the total number of byssal threads. The expression levels of all selected foot protein genes increased with byssus production but reached highest levels at different rates. Specifically, *HSP-70* reached its highest expression level on day 1 and maintained this level from day 1 to day 7. The expression level of *BP-3* exhibited a sharp increase on day 1 and reached the highest level on day 5. The expression levels of *TUB, BP-1, FP-2*, and *BPLP-2* sharply increased since day 3 and reached their highest levels on day 3, day 7, day 3 and day 7, respectively. It was thus clear that the expression profiles of *BP-1, FP-2*, and *BP-3* were closely related to byssus regeneration, suggesting that these genes play key roles in byssus formation.

### Ion composition and content

All 13 metal ions were tested from foot tissue and byssal thread samples with similar percentages (Table 3). Ca^2+^, Mg^2+^, Zn^2+^, Al^3+^, and Fe^3+^ were the most abundant metal ions in foot tissue with the content order of Ca^2+^ (35.59 mg·g tissue^−1^) > Mg^2+^ (26.37 mg·g tissue^−1^) > Zn^2+^ (23.28 mg·g tissue^−1^) > Al^3+^ (3.48 mg·g tissue^−1^) > Fe^3+^ (1.65 mg·g tissue^−1^), while Ca^2+^, Mg^2+^, Zn^2+^, Al^3+^, and Fe^3+^ were the most abundant metal ions in byssal threads with the content order of Mg^2+^ (3.81 mg·g byssus^−1^) > Ca^2+^ (3.58 mg·g byssus^−1^) > Al^3+^ (1.16 mg·g byssus^−1^) > Fe^3+^ (0.92 mg·g byssus^−1^) > Zn^2+^ (0.32 mg·g byssus^−1^).

**Table 3:** Metal ion contents in foot tissue and byssus of the golden mussel *Limnoperna fortunei* as detected by inductively coupled plasma mass spectrometry.

**Organ**	**Metal ion content (Foot: mg·g tissue^−1^/Byssus: mg·g byssus^−1^) and percentage**						
	**Ca^2+^**	**Mg^2+^**	**Zn^2+^**	**Fe^3+^**	**Al^3+^**	**Mn^7+^**	**Pb^4+^**
Foot	35.59 ± 3.55	26.37 ± 3.29	23.28 ± 2.00	1.65 ± 0.34	3.48 ± 0.48	0.15 ± 0.23	(33.54 ± 4.21) × 10^−3^
Percentage	38.80%	28.75%	25.38%	1.80%	3.79%	0.16%	0.04%
Byssus	3.58 ± 0.44	3.81 ± 0.24	0.32 ± 0.05	0.92 ± 0.13	1.16 ± 0.28	0.16 ± 0.02	(20.40 ± 3.55) × 10^−3^
Percentage	34.09%	36.25%	3.02%	8.76%	11.02%	1.55%	0.15%
**Organ**	**Metal ion content (Foot: mg·g tissue^−1^/ Byssus: mg·g byssus^−1^) and percentage**						
	**Cu**^2+^	**Hg**^2+^	**B**^3+^	**Mo**^6+^	**Ni**^2+^	**Sr**^2+^	
Foot	0.08 ± 0.01	0.13 ± 0.03	0.13 ± 0.04	(2.14 ± 0.42) × 10^−3^	0.42 ± 0.36	0.41 ± 0.13	
Percentage	0.09%	0.14%	0.15%	<0.01%	0.46%	0.45%	
Byssus	0.08 ± 0.01	(2.18 ± 0.53) × 10^−3^	0.31 ± 0.03	(1.36 ± 0.57) × 10^−3^	0.06 ± 0.01	0.09 ± 0.02	
Percentage	0.76%	0.02%	2.92%	0.01%	0.55%	0.89%	

## Discussion

Byssus morphology is largely similar among bivalves, but its ultrastructure can greatly vary among these species. In this study, byssal threads of the golden mussel *L. fortunei* became increasingly less compact from the proximal end to the distal end, which was consistent with previous studies on *D. polymorpha* (Farsad and Sone, 2012), *M. edulis* and *M. galloprovincialis* (Suhre et al., 2014; Reddy and Yang, 2015). This structure is important for mussel's self-reducing behavior, largely improving migration and predator-prey escape abilities by byssus de-adhesion and re-adhesion (Waite, 2017). The compact proximal end allows large numbers of byssal threads secretion in limited areas of foot tissues, and the less compact distal end facilitates inner fibers to spread out to form larger adhesive plaques, which may be both beneficial for byssus production and adhesion. Highly oriented structures in distal threads were considered to be related to byssus extensibility and stiff (Gosline et al., 2002; Farsad and Sone, 2012), representing firm byssus adhesion in *L. fortunei*. In addition, SEM results illustrated a highly porous adhesive plaque in *L. fortunei*, and such a structure was also observed in other mussel species such as *D. polymorpha* (Farsad and Sone, 2012), *M. edulis* (Benedict and Waite, 1986), and *M. californianus* (Waite, 1986). The number of cavities on adhesive plaque was suggested to be associated with adhesion ability as such cavities could participate in the formation of foot proteins by regulating protein coacervation (Waite et al., 2005). This view was supported by empirical evidence, for example, *M. californianus* has more pores in adhesive plaques than *M. edulis*, because the former lived in more turbulent intertidal environments (Waite, 1986). The abundant cavities may be conducive for *L. fortunei* to rapidly adhere and adapt to a new underwater environment. It is thus clear that the well-formed byssus structure of *L. fortunei* may increase invasive and fouling risks.

Structural integrity and adhesive properties of byssus are determined by foot proteins. Typical mussel adhesion-related foot proteins contain foot/byssal proteins (Mfps), collagen gland proteins (proCOLs) and thread matrix proteins (TPMs, Waite, 2017). In this study, the homologous genes coding these foot proteins were obtained from *L. fortunei* foot transcriptome. Sequence analyses indicated that these genes have conserved adhesion-related domains, such as EGF domain in *FP-2* and GXX domain in preCols. So far, more than six foot/byssal protein genes have been identified to be responsible for byssus adhesion, while only three of them (*BP-1, FP-2*, and *BP-3*) were isolated from *L. fortunei*. Interestingly, these *L. fortunei* foot protein genes showed low similarities to those found in other mussels. All results suggest a high level of species-specificity of these genes in bivalves. Indeed, adhesion-related proteins were diverse among aquatic organisms and even among bivalves (Miao et al., 2015), and most of them have more than one homologous genes. Such high polymorphism was suggested to explain the outstanding adaptabilities of mussels to a variety of substrata (Warner and Waite, 1999), which may be crucial to successful invasions and fouling by *L. fortunei*. Compared with other marine mussels, the foot/byssal proteins of *L. fortunei* were deficient in LC-MS/MS results, where only Lfbp-1, Lffp-2, and Lfbp-3 were identified, further demonstrating the high diversity of foot proteins in bivalves. In addition, proCOLs and TMPs were also absent in foot and byssus proteomes of *L. fortunei*. Collectively, three possible reasons may explain the absence of these proteins: (1) A major problem may be sample preparation, since the total foot tissue but not a limited region around the foot gland was used as the sample to conduct transcriptome sequencing. The whole foot sample can generate abundant transcripts to cover the relative low transcription of those genes for byssus synthesis and adhesion (Zhang et al., 2017). (2) These foot proteins may be different from known ones in structure and/or amino acid composition, thus it is difficult to be dissolved into the extraction solutions. (3) The widely distributed repetitive motifs may disturb protein enzymolysis and subsequently data search. Similar issues have been found in *M. coruscus* and *C. farreri* when their foot/byssal proteins were extracted (Miao et al., 2015; Qin et al., 2016). Acid-Urea methods were widely used by these studies to extract proteins from byssus because the generally major proteins in byssus (i.g. cross-linked proteins) were not simply isolated. However, this study confirmed that acetone precipitation method may be also effective in extracting foot and byssal proteins from mussel byssus. In addition, NBT/ Glycinate staining indicated that Dopa was located both in byssal thread and adhesive plaque of *L. fortunei*. The Dopa location regions of this species were consistent with that of the marine mussel *M. coruscus* (Qin et al., 2016), suggesting the basic characteristic of mussel Dopa-containing foot proteins.

By comparing foot transcriptome, foot proteome and byssal proteome, a total of 16 representative foot proteins and their coding genes were identified from *L. fortunei*. The homologs of these foot proteins have been proven to participate in byssus formation and interface adhesion in marine bivalves (Gantayet et al., 2014; Liu et al., 2015; Miao et al., 2015; Waite, 2017). The 16 proteins possessed abundant repetitive motifs. Motifs and modifications are the common features of adhesion-related proteins in marine mussels and scallops (Miao et al., 2015; Qin et al., 2016). Repetitive motifs often form regular or specific secondary structures (Taylor et al., 1994), which could promote interactions among foot proteins in *L. fortunei*. COG analysis indicated that the “Post-transcriptional modification, protein turnover, and chaperone” term was significantly enriched in foot transcriptome of *L. fortunei*. Modification plays a key role in byssus adhesion, for example, oxidation of Dopa (i.e., Dopa to dopaquinone) weakened adhesion of Mfps to substratum surfaces (Nicklisch et al., 2016), but strengthen cross-links among foot proteins during plaque formation (Yu et al., 2011). Staining analysis suggested that Dopa-containing proteins were located in byssal thread and adhesive plaque, which strongly supports the COG result. Carbamidomethyl modification, however, has not been well-studied on its specific function in regulating byssus adhesion though it was also identified in *C. farreri* byssus (Miao et al., 2015). Interestingly, COG and KEGG analyses indicated that the annotated unigenes in *L. fortunei* foot transcriptome were significantly enriched in some signal transduction pathways. These results obtained here may provide valuable points to further study signal regulations of byssus adhesion, as the detailed molecular regulatory mechanisms for byssus adhesion in aquatic organisms remain elusive (Miao et al., 2015).

The three representative foot proteins, Lfbp-1, Lffp-2, and Lfbp-3 located at foot tissue, were highly expressed in the rapid byssus formation period, suggesting their crucial functions during byssus production. Two homologous proteins of them, bp-1 and bp-3, have been reported in *M. coruscus* but their functions have never been illuminated (Qin et al., 2016). Therefore, particular attention has been paid to Lffp-2 because the functions of its homologous proteins have been well-studied in marine mussels. The foot protein fp-2 was firstly identified from adhesive plaque in *M. edulis* (Reddy and Yang, 2015). It is the most abundant foot protein in inner part of adhesive plaque with a molecular weight of ~45 KDa and a Dopa content of ~5 mol%, accounting for 25% wet weight of plaque. It contains eleven EGF domains with abundant Ca^2+^ binding sites. Several homolog genes/proteins of *FP-2*/fp-2 have been isolated from mussels, including Pffp-2 from *L. fortunei* (Hwang et al., 2010; Uliano-Silva et al., 2014; Qin et al., 2016). The Pffp-2 is the only identified foot protein for which the open reading frame (ORF) has been sequenced so far (Uliano-Silva et al., 2014). In this study, the conserved EGF domain was found in Pffp-2, suggesting the potential adhesive function of this foot protein in *L. fortunei*. RT-qPCR analyses also strongly indicated that *PfFP-2* may be crucial to the adhesion of adhesive plaque in *L. fortunei*. Moreover, the specificities of other foot proteins such as enzymes and cellular fragment proteins were not as strong as foot/byssal proteins in the present study. It was reported that artificial contamination in the process of protein extraction could result in the presence of these proteins in byssus proteome (Qin et al., 2016). Whether these proteins in *L. fortunei* byssus are contamination is worth further studying in the future. However, these proteins might be involved in byssus adhesion because they were identified not only in the byssus of *L. fortunei* but also in a variety of bivalves (Farsad and Sone, 2012; Gantayet et al., 2014; Miao et al., 2015; Qin et al., 2016).

The 16 representative foot proteins in *L. fortunei* have eight highly dominant amino acids. The abundances of these amino acids were between 6 and 18%, which were more than that in vertebrates (typically only 2–5% in vertebrate proteins), suggesting a high amino acid preference of these foot proteins. Such a high preference is similar to that found in freshwater mussel *D. polymorpha* (Gantayet et al., 2014) but dissimilar to that in marine mussel *M. oruscus* (Qin et al., 2016), suggesting the distinct amino acid preference between freshwater and marine species in foot proteins. Amino acids have strong metal ion binding capacity, for example, Glu and Asp most frequently bind to metal ions *via* charged or polar side chains (Golovin et al., 2005). Metal ions also can selectively bind to amino acids, for example, Ca^2+^ to Asn, Asp, Gly and Glu, Mg^2+^ to Glu and Asp, Fe^3+^ to His, Asp, Cys, Tyr and Glu, and Zn^2+^ to His and Cys (Lu et al., 2012). ICP-MS analysis demonstrated that Ca^2+^, Mg^2+^, Zn^2+^, Al^3+^, and Fe^3+^ were the most abundant metal ions in both foot tissue and byssal thread, which perfectly correspond to amino acid preference in the foot proteins of *L. fortunei*. Previous studies have found that mussel byssus and soft tissues had a strong ability to adsorb heavy metals, such as Ag, Ni, Mn, Fe, Zn, Hg, and Cd from surrounding environments by forming metal complexes between specific amino acids of foot proteins and heavy metals. Even after separation from mussels, the byssus still had a strong adsorption to these heavy metals (Szefer et al., 2002; Zhang et al., 2017). To a large extent, the detected high concentration of heavy metals in foot tissue and byssus in this study may be the result of metal accumulation by *L. fortunei*. In addition, metal ions such as Fe^3+^ and Ca^2+^ have significant impacts on the interactions between fp-2 and other foot proteins (e.g., Fp-3, Fp-4, and Fp−5) by binding to specific amino acid residues, forming the core function of adhesive plaques (Hwang et al., 2010; Lee et al., 2011). These metal ions also pose significant effects on self-assembly and Dopa oxidative cross-linking of adhesion-related proteins (Guo et al., 2015; Liu et al., 2016; Priemel et al., 2017). As mentioned above, Dopa has been observed in both byssal thread and adhesive plaque of *L. fortunei*. Therefore, we reasonably speculate that some metal ions play critical roles in byssus adhesion by interacting with amino acid residues in foot proteins of *L. fortunei*, which may also be the reason for the high concentration of other metal ions in byssus. (Taylor et al., 1994; Zhao and Waite, 2006; Harrington et al., 2009, 2010; Yu et al., 2011). More importantly, metal ions can affect byssus mechanical properties, as studies had shown that the strength of byssal threads varied with the metal ion concentrations in marine bivalves (Seguin-Heine et al., 2014; Li S. G. et al., 2017). These results illustrated the underlying roles of the interactions of metal ions and foot proteins in byssus adhesion, thus proving a valuable window of opportunity to manage biofouling through artificial intervention on these determining factors in either *L. fortunei* or other bivalve species such as *Dreissena* mussels (Gilbert and Sone, 2010; Gantayet et al., 2013; Rees et al., 2016).

## Conclusions

Using multiple approaches, this study represents the first effort to clarify byssus structure, identify foot proteins and determine metal ions of foot tissue and byssus in the golden mussel *L. fortunei*. The obtained results suggest the structural characteristics of adhesive plaque, proximal, and distal threads highly conducive to byssus adhesion. Dopa is proved to be a *post-translational* modification in byssal thread and adhesive plaque. Sixteen representative foot proteins and their coding genes were identified from *L. fortunei*. In addition to heavy metals, potential interactions of these identified proteins and some specific metal ions may be important for byssus structural integrity and adhesive properties, indicating the key roles of foot proteins and metal ions involved in byssus adhesion. These results are beneficial to further reveal the molecular mechanism of *L. fortunei* byssus adhesion. Owing to the significant environmental implications, a comprehensive understanding on the mechanisms of byssus adhesion in freshwater mussels will facilitate to make strategies against biofouling of aquatic invasive organisms. In addition, the identified proteins from *L. fortunei* largely enrich the members of foot protein families in freshwater bivalves, providing abundant available genetic resources for underwater adhesives and antifouling materials studies.

## Ethics statement

This study did not involve endangered species and no specific permit was required for sampling.

## Author contributions

SL and AZ designed and conducted the experiments. ZX, YG, and YC collected mussel samples. SL and AZ conducted data analyses and wrote the first version of this manuscript. SL, AZ, ZX, YG, and YC contributed to the completion of the manuscript.

### Conflict of interest statement

The authors declare that the research was conducted in the absence of any commercial or financial relationships that could be construed as a potential conflict of interest.
