# Supplementary material for: Byssus Structure and Protein Composition in the Highly Invasive Fouling Mussel Limnoperna fortunei
Source: Front Physiol. 2018 Apr 16;9:418. doi: 10.3389/fphys.2018.00418 (PMC5911496; doi:10.3389/fphys.2018.00418)
Supplement: Supplementary file 1 [file Table1.DOC]

**Byssus Structure and Protein Composition in the Highly Invasive Fouling Mussel *Limnoperna fortunei***

**Shiguo Li^1^, Zhiqiang Xia^1,2^, Yiyong Chen^1,3^, Yangchun Gao^1,3^ and Aibin Zhan^1,3*^**

^1^Research Center for Eco-Environmental Sciences, Chinese Academy of Sciences, Beijing, China; ^2^Great Lakes Institute for Environmental Research, University of Windsor, Windsor, Canada; ^3^University of Chinese Academy of Sciences, Chinese Academy of Sciences, Beijing, China;

***Correspondence**

Prof. Aibin Zhan.

E-mail: zhanaibin@hotmail.com; azhan@rcees.ac.cn.

**TABLES**

**TABLE S1** Primers used for real-time quantitative PCR. F: Forward primer. R: Reverse primer.

| Symbol | Gene ID | Gene description | Production size | Primers |
| --- | --- | --- | --- | --- |
| BP-3 | comp112372_c0_seq2 | gi|824631701|gb|AKI87986.1|  byssal protein-3 [*Mytilus coruscus*] | 95 | F: GTTCGCAAAGCTGTCGTGTT  R: CCAGGTCCAAATTTTGCGCA |
| HSP70 | comp113462_c0_seq1 | gi|76780606|emb|CAH04106.1|  heat shock protein 70  [*Mytilus galloprovincialis*] | 146 | F: CTGAAGCCTACCTCGGACAA  R: GGCGGCTGTTGGTTCATTTA |
| TUB | comp39206_c0_seq1 | gi|405965638|gb|EKC31000.1|  Tubulin alpha-1C chain [*Crassostrea gigas*] | 107 | F: ATCCACGTATCCACTTCCCC  R: CTCGAAGGTGGCATTTGTGA |
| BPLP-2 | comp125734_c0_seq1 | Byssal peroxidase-like protein 2  [Mytilus coruscus] | 137 | F: AAGAGACACAGCGGTAGGAC  R: ACGTTCTGGATGGTGGTTCT |
| BP-1 | comp98491_c0_seq1 | gi|824631657|gb|AKI87985.1|  byssal protein-1 [*Mytilus coruscus*] | 81 | F: TGCCAAGTGTCCGAAGTTTC  R: CCATCAACAAACCAAACGGC |
| FP-2 | comp119431_c0_seq1 | gi|60548046|gb|AAX23970.1|  foot protein 2 [*Mytilus edulis*] | 128 | F: TCCCTAACCCCTGCAAACAT  R: GTGACACGGATTAGGTTCGC |

**TABLE S2** Statistics for the transcriptome assembly. Q30: The percentage of the base number (Phred > 30) from raw data in the total bases.

| Term | Raw reads | Clean reads | Q30 |
| --- | --- | --- | --- |
| Reads | 73231128 | 73231128 | 92.89% |

**TABLE S3** **Statistics for the transcriptome assembly. N50: 50 % reads longer than or equal to the length of unigenes.**

| Term | All  (>300 bp) | >=500 bp | >=1000 bp | N50 | Total Length | Maximum  Length | Minimum  Length | Average  Length |
| --- | --- | --- | --- | --- | --- | --- | --- | --- |
| Unigene | 92,951 | 36,051 | 16,081 bp | 946 | 63,298,947 bp | 13,793 bp | 251 bp | 680.99 bp |

**TABLE S4** COG enrichment of the unigenes in foot transcriptome of the golden mussel *Limnoperna fortunei.*

**See supplemental Excel Table S4.**

**TABLE S5** KEGG enrichment of the unigenes in foot transcriptome of the golden mussel *Limnoperna fortunei.*

**See supplemental Excel Table S5.**

**TABLE S6** The most representative foot proteins identified from byssal thread of the golden mussel *Limnoperna fortunei*.

**See supplemental Excel Table S6.**

**TABLE S7** The most representative foot proteins identified from foot tissue of the golden mussel *Limnoperna fortunei*.

**See supplemental Excel Table S7.**

**FIGURES**


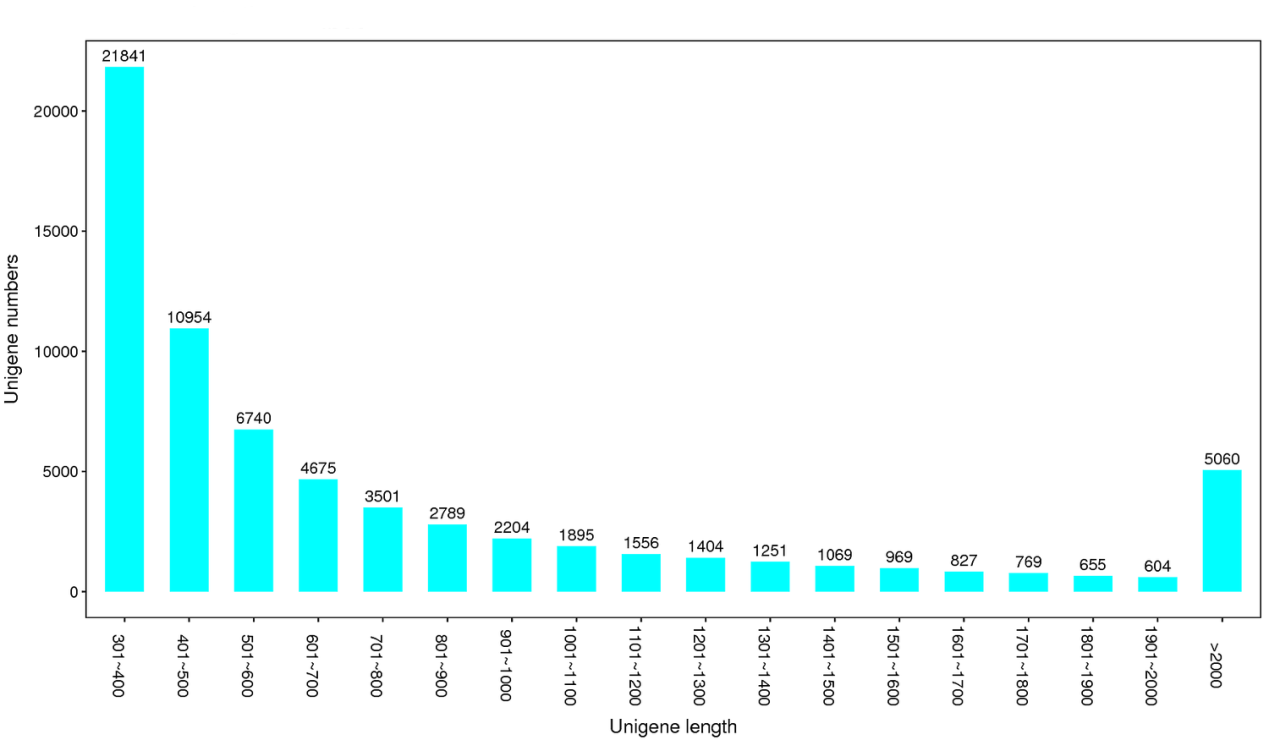


**FIGURE S1** Length distribution of the unigenes in the foot transcritome of the golden mussel *Limnoperna fortunei.*


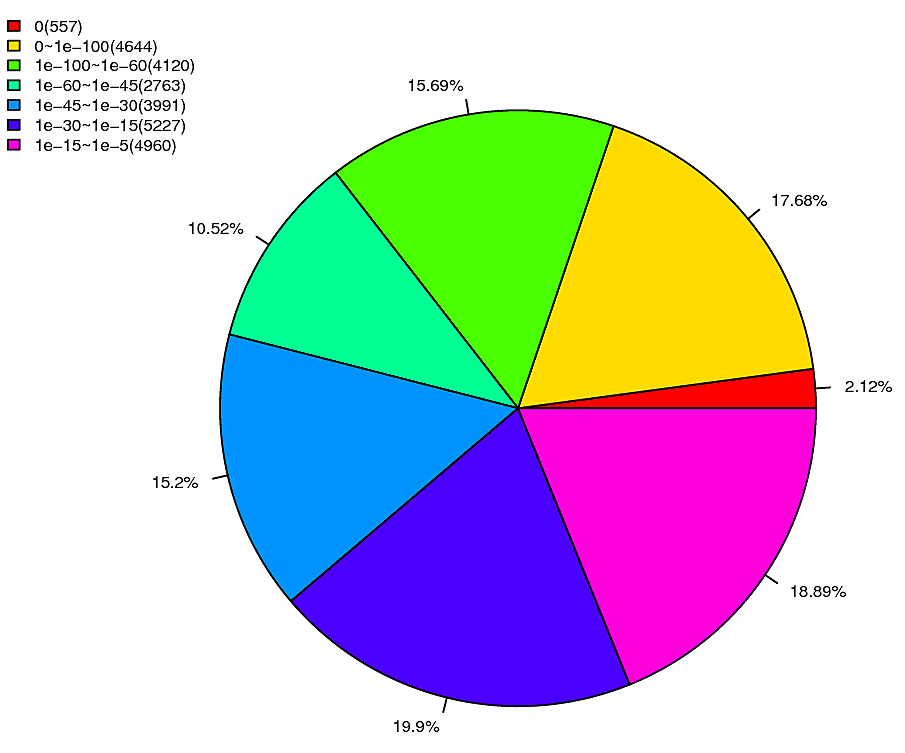

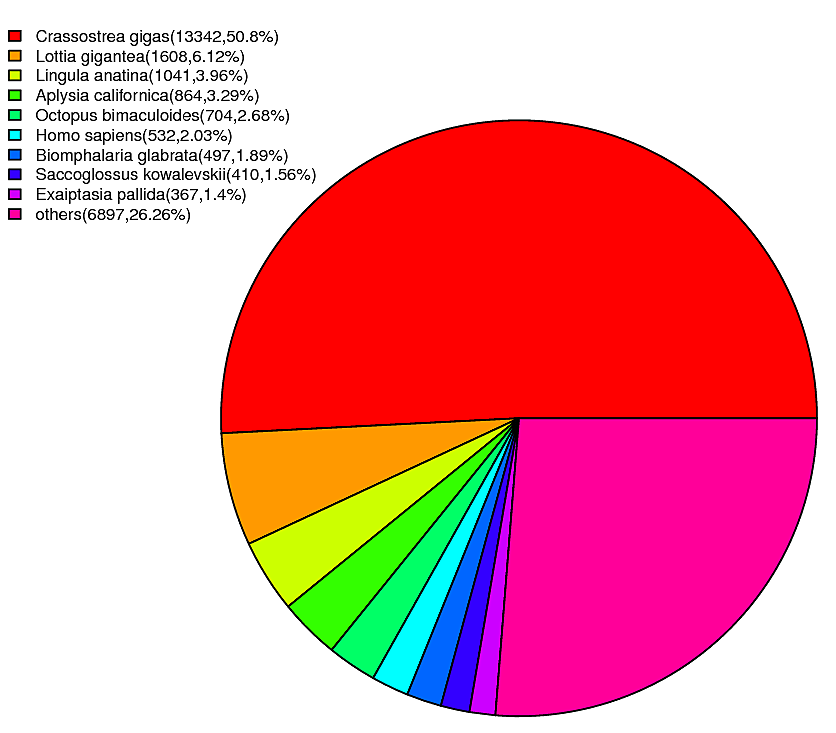


E-value distribution

Species distribution

**FIGURE S2** E-value and species distributions for the unigene annotated against NR database.


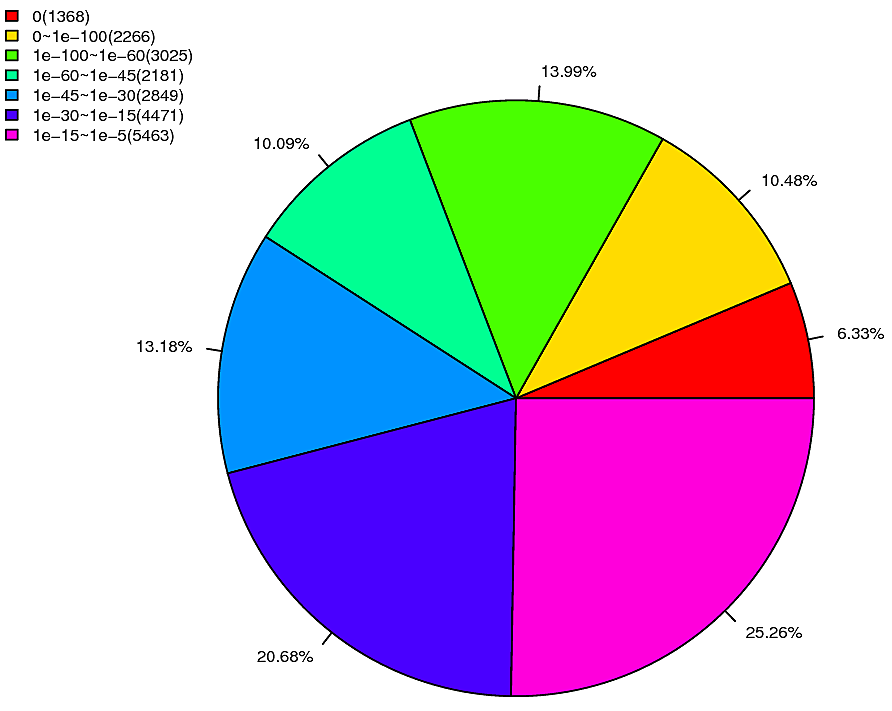


E-value distribution


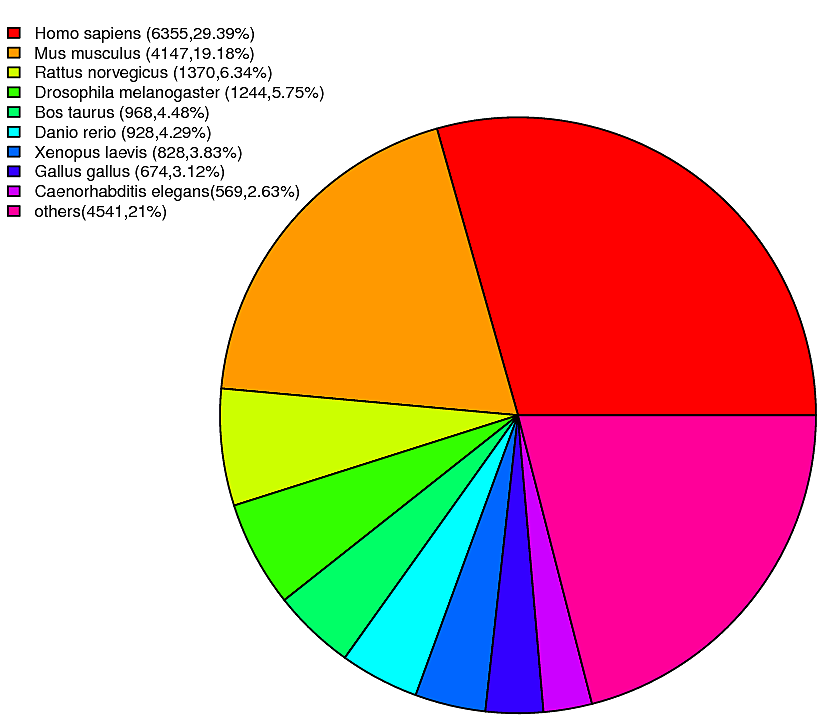


Species distribution

**FIGURE S3** E-value and species distributions for the unigene annotated against Swiss-Prot database.

**
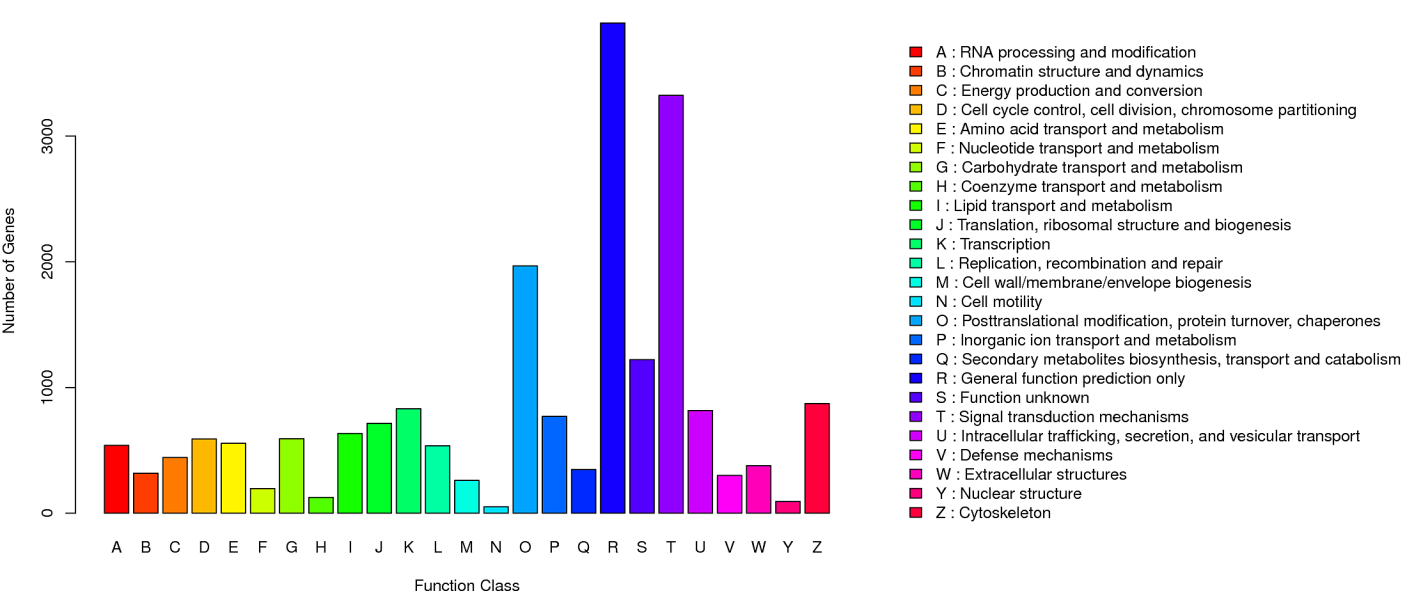
**

**FIGURE S4** COG annotation for the foot transcriptome of the golden mussel *Limnoperna fortunei*. A total of 17,871 unigenes with significant homologies are assigned into 25 COG categories (E-value *≤* 1.0e-5).


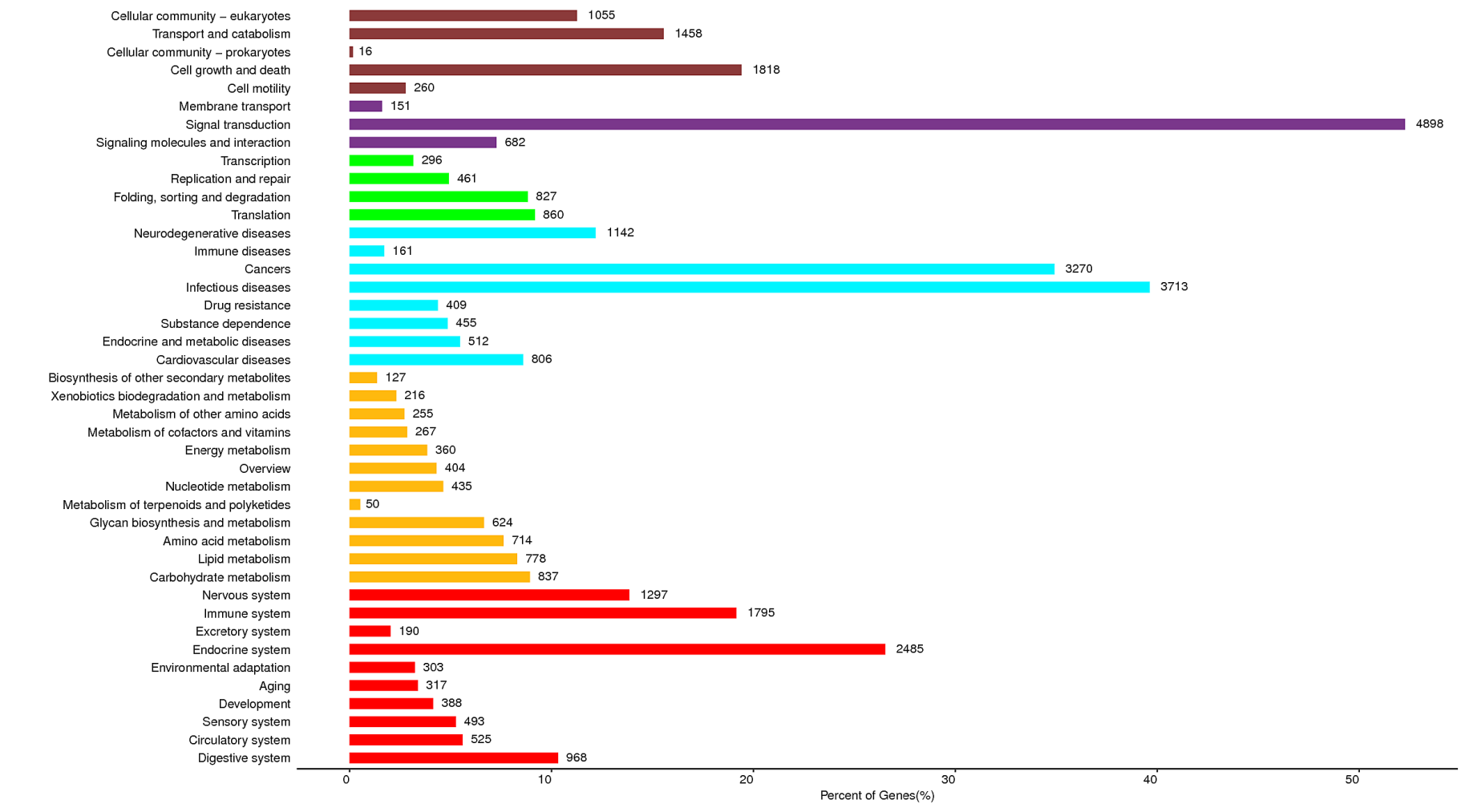


**FIGURE S5** KEGG annotation for the foot transcriptome of the golden mussel *Limnoperna fortunei*. A total of 9,368 unigenes with significant homologies are assigned into 42 KEGG categories (E-value ≤ 1.0e-5).


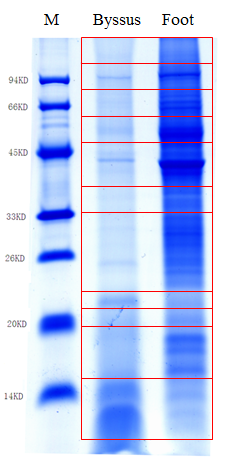


**FIGURE S6** Sodium dodecyl sulfate polyacrylamide gel electrophoresis (SDS-PAGE) of the proteins extracted from foot tissue and byssal threads of the golden mussel *Limnoperna fortunei*. The red boxes indicate the selected regions used for mass spectrometric analysis. M: Markers for protein molecular weight.


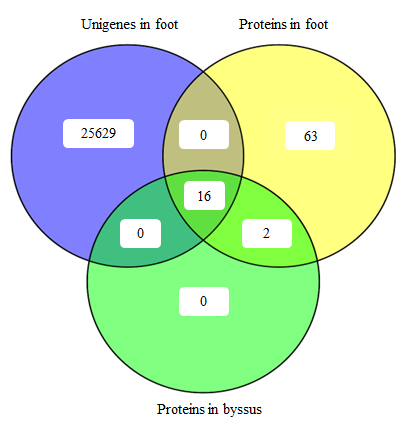


**FIGURE S7** Venn diagram showing the most representative foot proteins identified from foot transcriptome, foot proteome and byssal proteome of the golden mussel *Limnoperna fortunei*.
